# The problem of value change: Should advance directives hold moral authority for persons living with dementia?

**DOI:** 10.1111/bioe.13386

**Published:** 2024-12-15

**Authors:** Anand Sergeant

**Affiliations:** ^1^ Departments of Philosophy and Continuing Education University of Oxford Oxford UK; ^2^ Schulich School of Medicine Western University London Canada

**Keywords:** advance directives, decision‐making, dementia, end‐of‐life care

## Abstract

As the prevalence of dementia rises, it is increasingly important to determine how to best respect incapable individuals' autonomy during end‐of‐life decisions. Many philosophers advocate for the use of advance directives in these situations to allow capable individuals to outline preferences for their future incapable selves. In this paper, however, I consider whether advance directives lack moral authority in instances of dementia. First, I introduce several scholars who have argued that changes in peoplewith dementia's values throughout disease progression reduce the validity of their advanced wishes. I then outline Karin Jongsma's rejection of this claim, which she calls the “losing and choosing” distinction. Jongsma argues that changes in people with dementia's values should not be respected, because they are unchosen and dictated by the disease. I critique her claim that the process of value change is morally relevant when determining which values we respect. I argue that if individuals with dementia are capable of valuing, their contemporary values should be respected, even when they conflict with past preferences outlined in an advance directive. As such, situations of value change diminish the moral authority of advance directives for individuals with dementia.

## 1 INTRODUCTION

As our population ages, an increasing number of individuals will be afflicted by age‐related disorders such as dementia—a group of conditions characterized by progressive worsening of a person's cognitive abilities.^1^ Typically, dementia develops in older individuals, who first experience memory difficulties followed by impairments of other cognitive functions.^2^ As of 2019, 57.4 million individuals had dementia globally; this number is expected to surpass 150 million by 2050.^3^

As dementia progresses, individuals become more dependent on caregivers as their cognitive capacities diminish. This makes it crucial to determine ways to make end‐of‐life decisions that optimally align with patients' preferences. Generally, individuals who are losing decision‐making capacity choose or are assigned a substitute decision‐maker. However, many scholars have advocated for the use of advance directives for dementia care.^4^ An advance directive is a document that allows a capable individual to outline preferences for their future self who is incapable of making these decisions.^5^ Advance directives are legally binding in many countries and allow patients to make a variety of medical decisions.^6^ In the Netherlands and Belgium, advance directives can be made for decisions as weighty as euthanasia, and similar legislation efforts are predicted elsewhere.^7^

In this paper, I examine whether advance directives lack moral authority when applied to individuals with dementia. First, I discuss the importance of autonomy in grounding the moral authority of advance directives. Next, I introduce arguments that propose that the occurrence of preference changes in people with dementia places the authority of advance directives in doubt. I then outline a rebuttal to this position made by Karin Jongsma, which she terms the “losing and choosing” distinction. My objective is to critique Jongsma's claim that the unintentional nature of a person with dementia's value change diminishes the moral weight of their new preferences. I argue that people with dementia's changing values should be respected, which diminishes the moral authority of advance directives.

## 2 THE MORAL AUTHORITY OF ADVANCE DIRECTIVES

Various arguments have been made to assert the moral authority of advance directives. One holds that advance directives hold moral value because they produce decisions in patients' best interests. As Buchanan and Brock describe, allowing individuals to self‐determine is “instrumentally valuable in promoting a person's well‐being.”^8^ Advance directives thus hold prudential moral authority by allowing individuals to judge their future interests. The argument for best interests is also supported by Dworkin.^9^ Dworkin claims that advance directives allow us to respect individuals' “critical interests”—interests concerning the overall success of their lives—which hold more prudential value than day‐to‐day “experiential interests.”^10^

Dworkin, moreover, argues that advance directives hold moral weight because they best respect individuals' autonomy.^11^ Dworkin argues that our reasons for respecting individuals' choices go beyond their instrumental value. This is evidenced when we respect individuals' decisions even when they make choices that are harmful to their well‐being—for example, individuals who smoke despite understanding that it is against their best interest. Dworkin argues that we need a stronger account of the value of autonomy, which he labels the “integrity view.”^12^ His “integrity view” holds that the value of autonomy “derives from the capacity it protects: the capacity to express one's own character.”^13^ This view explains why we allow individuals to lead lives according to their distinctive personalities and values, even if they make decisions that may seem contrary to their best interests.

The integrity view requires individuals to have a “coherent sense of self” to make decisions that truly reflect their deepest convictions.^14^ This raises a challenge for the treatment of individuals with dementia because many of these individuals lose the capacity to retain coherent identities. While this implies that these individuals lose their autonomy rights, Dworkin argues we should respect people with dementia's “precedent autonomy” under the integrity view. If an individual with dementia cannot exercise fresh autonomous claims, Dworkin proposes that their previous, coherently formed decisions should be respected.

The notion of precedent autonomy is accepted by many philosophers and represents the conceptual foundation for the moral authority of advance directives.^15^ In cases of incompetence, such as Dementia, advance directives hold authority as a means of respecting individuals' autonomy by prioritizing their carefully formed life preferences. This justification for advance directives will be the focus of this paper.

## 3 VALIDITY AND VALUE CHANGE

The belief that an individual's precedent autonomy should extend to their future self is controversial. Instances in which people with dementia's current wishes seemingly oppose their previous preferences, as outlined in Dworkin and Jaworska, make it unclear whether advance directives hold validity in cases of dementia.^16^

For example, Dworkin discusses the case of Margo, an Alzheimer's disease patient described by Firlik.^17^ Margo has advanced dementia; she listens to the same songs repeatedly and does not remember the names of her caretakers. Nonetheless, Firlik describes Margo as “one of the happiest people [he] has ever known”—she is cheerful and enjoys engaging in experiences such as reading and painting.^18^ Dworkin asks us to imagine that years prior to the onset of her Alzheimer's disease, Margo expressed that she would not want her life to be extended if she developed dementia.^19^ Should this directive bear weight and potentially end her happy life if she requires medical treatment for a reversible condition?

One important criticism of the validity of such advance directives focuses on the numerical identity of individuals with dementia—*who* the person with dementia is.^20^ Some scholars argue that losses of cognitive capacities characterized by dementia may cause an individual to become a completely different person, undermining the validity of advance directives.^21^ These accounts typically appeal to theories of identity that ground personal identity in psychological continuity; they argue that dementia changes an individual's numerical identity as they lose psychological connection with their former self.^22^ While more work is needed on this matter, I assume in this paper that individuals with dementia remain the same numerical person throughout the disease. Here, I follow Rhoden, who holds that a complete conception of identity must take both an internal and historical view, which considers a person's identity in the context of a larger life.^23^ A full view of personhood must consider an individual as a person with a life history within a web of relationships that persist even when their capacity fails. Individuals with dementia can thus remain the same numerical person despite memory failures which disrupt their psychological continuity. This view aligns with our treatment of persons with dementia's numerical identity clinically, legally, and socio‐culturally.

The concerns focused on in this paper have to do with the qualitative identity of individuals with dementia—*how* they are, including their character, values, and preferences, and how these change over the disease course.^24^ If the objective of an advance directive is to extend an individual's choice to their future self, a valid advance directive should make judgments that align with the contemporaneous values of the individual with dementia. However, critics doubt whether this is the case. In her “transformative experience” argument, Walsh suggests that the gradual alteration of individuals' capacities over the course of dementia may induce preference revelations over time, due to the cognitively transformative experience of the disease.^25^ This presents a challenge to the authority of advance directives because if an individual's values change during the dementia process, it is possible that an advance directive does not adequately reflect those of the later individual and could contradict their preferences at a time when they are deemed incapable of expressing them.^26^ Jaworska asserts that individuals with dementia can indeed value, and these values should be respected as they change through the dementia process, even if the patient is deemed medically incapable.^27^

Moving forward, I will use the phrase “value change” to refer to changes in an individual's overall set of values. This can occur either through changes in an individual's values themselves, such as the development or deprioritization of a value, or due to changes in the relative weight held by a given value compared to others—for instance, when one value is diminished or forgotten through memory loss, another may take priority. The possibility of value changes jeopardizes the moral authority of advance directives, by calling into question the validity of extending an individual's prior wishes to a future point in which they may hold different priorities.

## 4 CHOOSING VERSUS LOSING—JONGSMA'S REBUTTAL

Jongsma rejects the notion of value change in the context of dementia.^28^ To Jongsma, patients with dementia “do not *choose* what they forget; therefore, what they still remember and the values they still express are not to be considered the ones with the highest priority.”^29^ Since the contemporary values of individuals with dementia are dictated by their disease rather than by a conscious choice to change values, Jongsma argues that their remaining values should not be prioritized over their previously expressed preferences.^30^ Jongsma labels this the “losing and choosing” distinction.^31^ She gives the example of an athlete who chooses to relinquish his value of running and develop new values once he loses his ability to run in a tragic accident.^32^ While a person with dementia might similarly act as though they have dropped their former values, Jongsma denies that they should be treated the same as the runner. According to Jongsma, individuals who drop or change values due to unchosen factors such as cognitive decline should not be treated equivalently to those who choose to change which values are most important to them.^33^

In the rest of this paper, I argue that the “choosing versus losing” distinction fails to explain why patients' current values, if they exist, should be outweighed by prior directions.

## 5 DEMENTIA AND THE CAPACITY TO VALUE

The argument from value change assumes that patients with dementia can indeed change their values and preferences. This means that patients deemed incapable of changing their advance directive can still have changes in their preferences that they are unable to articulate under the standards required for capacity.

Some, like Jongsma, question this assumption and claim that individuals with dementia are incapable of valuing in the manner necessary for them to override an advance directive. Jongsma and colleagues distinguish between preferences—which are driven by underlying values developed over time—and “wishes,” which are more transient desires arising moment‐to‐moment.^34^ They claim that while people with dementia can have wishes, it is unlikely that they have the capacities necessary to value. Thus, they are not able to generate novel preferences regarding their care.^35^

However, I believe this claim is premature. The notion that persons with dementia cannot value depends on one's definition of valuing, and it is not clear that, as Jongsma asserts, values are inclinations that must be developed, reflected upon, and adjusted over time.^36^ For instance, in an alternative conception of valuing, Jaworska argues that while persons with dementia may experience memory impairment, their ability to value can remain intact as they can make judgments regarding what is best for them grounded in a set of normative ideals.^37^ Jaworska gives an example of a patient who cannot name the day of the week, form new memories, or find her way to the bathroom; however, this patient is nonetheless able to value—such as when she expresses a preference to volunteer as a research subject because she believes she should “help her fellow man.”^38^ In another prominent account of valuing, Scheffler requires an individual to believe something is valuable, view this thing as providing reasons for action, and experience a range of emotions toward it that the person believes are appropriate.^39^ It is likely that many individuals with dementia, such as the one in Jaworska's example, can fulfill these criteria, despite having diminished cognitive capacities. Even individuals such as Margo, who enjoy elements of their life such as speaking with loved ones, and place importance on these elements by pursuing them regularly, can be said to value under this model.^40^

Moreover, the existing empirical evidence suggests that the ability to value and generate preferences may be present in many cases of moderate dementia. Smebye et al. describe how many individuals with dementia, despite having diminished cognitive abilities, can have their values clarified through discussion with caregivers to empower these individuals to engage in varying degrees of decision‐making.^41^ Furthermore, numerous studies have found that individuals with progressing dementia can adopt strategies to retain control over their lives, including focusing on the present amidst memory loss,^42^ which can allow these individuals to generate preferences based on their contemporaneous values. This evidence provides support for a definition of valuing which is independent of long‐term memory and reflection, contrary to Jongsma's account of valuing, which suggests that it is possible for individuals with dementia to generate preferences based on underlying values, even when it is difficult to articulate them.

Importantly, this does not imply that individuals with moderate‐to‐severe dementia retain full decision‐making capacity. Medical capacity refers to whether an individual has the required abilities to make an independent decision.^43^ In the medical context, capacity depends on various factors, such as an individual's verbal fluency, their ability to hold and manipulate information using their working memory, and their ability to value and weigh choices, which can all be impaired by neurodegenerative diseases such as dementia.^44^ Competency, a related concept, is a legal determination of whether an individual's impaired capacity should limit their ability to make a legally pertinent choice, such as decisions related to end‐of‐life care.^45^ What is crucial to note is that while the ability to value is one element of decision‐making, it is not the same concept as decision‐making capacity or legal competence. This means that an individual with declining neuro‐cognition may be able to retain the ability to value, even though they may have lost decision‐making capacity, through, for instance, declining verbal fluency, working memory, or task‐setting abilities.

More work is needed on our definition of what it means to value, and the neurophysiological underpinnings for this capability.^46^ It is unclear exactly at which point an individual's ability to value degenerates, and how closely this aligns with the loss of medical capacity. Given the empirical evidence, however, it is reasonable to assume that, at least in some cases, individuals with advanced dementia can carry the ability to value past the stage at which they are deemed medically incapable.^47^

## 6 THE VALUE OF UNCHOSEN PREFERENCES

Jongsma argues that because individuals with dementia do not choose what they forget, their current values should not be prioritized compared to the capacious preferences outlined in their advance directive. This means that if an individual with dementia has a seeming shift in their preferences, it should be disregarded because it was unchosen and due to the disease.

The choosing versus losing argument assumes that there is a morally relevant difference between values that are chosen and values that are a result of factors outside of someone's control. However, there are several problems with this assumption. First, it is unclear what constitutes “chosen” values. Our values develop from numerous sources—our parents, our education, the people we happen to meet, and our varying lifetime experiences—most of which are outside of our control. Individuals are not blank slates who, only after long periods of critical reflection, decide which values to choose. Individuals accept values arising from a variety of unchosen circumstances, including societal norms and social influences, and in these situations, we still respect individuals' values and ascribe autonomy to their related preferences. We respect a person's values not because they have “chosen” them but because they affirm them and take them as their own. If we accept that, in some cases, individuals with moderate‐to‐severe dementia who are deemed incapable can still value, we should not override these values simply because they arose from an unchosen disease. The mere fact that they can express and affirm certain convictions carries moral weight when we are deciding how to best respect their preferences.^48^

Second, while the choosing versus losing argument implies that unchosen preferences should not hold moral authority over previous, chosen preferences, there are many cases in which we ought to respect current preferences, despite them being seemingly unchosen. For instance

Carl is a 60‐year old school teacher with a family history of Alzheimer's disease. When he was a teenager, he took care of his grandmother, Diana, as she progressed through the various stages of dementia. They enjoyed a good relationship, until Diana's dementia reached an advanced stage, at which point she no longer recognized Carl. Following Diana's death, Carl decided that he never wanted to reach the point of life where he ceased to recognize his loved ones. Thus, Carl told his physician that if he ever reached that stage of dementia, he would not like to receive life‐extending care. Unfortunately, a few years later, Carl gets into a dangerous car crash. While he survives, the crash causes Carl to develop memory impairments, such that Carl retains no memory of his grandmother's experience with dementia. Because these memories are inaccessible, he no longer feels his ability to recognize family members should be a relevant factor regarding his end‐of‐life care. He expresses this to his physician when he is reminded of his previous wishes.

Carl, in Jongsma's words, “did not choose what he [forgot],” nor did he choose the preference changes that arose due to his memory loss.^49^ Yet, it is clear in this case that we should respect Carl's new preferences. The mere fact that this value change was unchosen does not imply that his values should be overridden by previous “chosen” values developed from his experiences with his grandmother. Given his memory loss, Carl's value of recognizing his loved ones was diminished, and other values, such as his value of physical comfort and enjoying his remaining experiences, took precedence within his existing value set. That Carl affirms new values and prioritizes them over his previous values is what is relevant to our decision on how to respect his autonomy.

One might object by arguing that a difference between Carl's case and an individual with dementia is that the latter may lose other cognitive abilities, making them medically incapable of changing their advance directive and making well‐reasoned decisions regarding end‐of‐life care. However, if we accept that individuals with dementia can retain the capacity to value, then we should be wary of the possibility that their pre‐dementia preferences have changed as they lose memories and emotional connections that informed their past decisions.

For instance, suppose that Carl did not get into a car accident. Instead, a few years later, Carl is diagnosed with Alzheimer's. As his disease progresses, Carl loses significant memory, including that of his grandmother. While he affirmed his advance directive earlier in the disease course, Carl no longer indicates any fear regarding his dementia. He expresses enjoyment of his life and seems to value his day‐to‐day experiences as he socializes with fellow patients, listens to the birds, and reads the newspaper. Carl affirms his value of continued life when he expresses that “things are simpler now, but I've learned to enjoy the experiences I have left.”

Like the last time, Carl's loss of memory is unchosen: it is completely dictated by the course of the disease. However, Carl is still able to value his remaining experiences. While he may be deemed incompetent and unable to communicate these preferences in the context of his end‐of‐life care, there is clearly a potential conflict between his previous conviction that a life without recognition of family members would not be worth living and the value that he currently places on his remaining existence. Previously, Carl may have been fearful of a life where he lacked the ability to engage in complex cognitive activities, hence the creation of his advance directive. However, similar to the case of the car‐accident, the fact that the loss of this end‐of‐life preference was unchosen should not be morally relevant, so long as he retains the capacity to value other things, which now trump his previously lost values in priority. In this case, Carl's values of comfort and enjoyment of daily experiences, perhaps lower on his previous value hierarchy, are now prioritized because they are the values that remain. Respecting these remaining values is the best way to respect an individual with cognitive decline like Carl because it acknowledges that individuals' priorities can change over the course of dementia, as they lose cognitive complexity and connections to their former self.

## 7 VALUE CHANGES AND THE ROLE OF AUTONOMY

Even if we should be wary of the possibility of value change in incompetent individuals, some might contend that current autonomy is necessary for the new values to hold moral significance. Unlike the first case, when post‐accident Carl retains the autonomy and decision‐making capacity to affirm his change in values, many individuals with dementia are not autonomous decision‐makers. Perhaps the central concern with respecting value changes in individuals with dementia has nothing to do with the unchosen process of these value changes. Rather, value changes should not be respected because the current individual lacks autonomy, and autonomy is necessary for an individual's current values to overturn previous, competently formed preferences. If the current individual is non‐autonomous, as is often the case in moderate‐to‐severe dementia, then perhaps their changing values should not hold any moral significance and we should defer to their former preferences.

The argument from current autonomy fails to explain why the current values of individuals with dementia should not be respected. For one, it asserts a firm dichotomy between non‐autonomous decision‐makers and autonomous decision‐makers, which does not adequately reflect the nuanced reality of cognitive decline within dementia. There is no singular point at which an individual with dementia loses their autonomy. An individual with dementia can be autonomous with respect to one decision but deemed non‐autonomous with respect to others, and, as I described earlier, individuals who are deemed incapable can engage in varying degrees of decision‐making. Autonomy exists on a continuous scale, and individuals with neurocognitive decline lose their autonomy gradually, even once medical professionals deem them incapable. It is thus problematic to assume that individuals either have autonomy and their values should be respected, or that they do not, and their values should be disregarded. Many individuals with dementia have varying degrees of diminished or “partial” autonomy, which should be fostered as much as possible.

The notion of “partial” autonomy can be understood in terms of some of the most widely accepted accounts of autonomy used in medical ethics. For example, with respect to decision‐making, Beauchamp and Childress' account of autonomy requires an autonomous decision to fulfill three conditions: understanding, voluntariness, and intentionality,^50^ and all of these conditions can be understood as existing on a continuous scale.^51^ Individuals can understand the meaning and consequences of a decision to different degrees, can be more or less in control of their decisions, and can engage in varying degrees of goal‐setting and planning.^52^ This also applies to individuals with dementia. Depending on the stage of the disease and the decision being made, individuals with dementia can understand the implications of a decision to varying degrees, be more or less in control of their decision, and engage in various degrees of intentionality by creating sub‐plans and acting in ways which correspond to them.^53^ They therefore have a wide possible range of autonomy, which continues even once they are deemed incapable, as formal assessments of capacity set thresholds, often arbitrarily, upon the continuous attribute of autonomy.

The notion of partial autonomy is crucial because it means that many individuals with dementia who have lost the ability to make fully autonomous decisions still retain present autonomy considerations. This can be seen in other instances of diminished autonomy within medical decision‐making, such as medical decision‐making in young children, where kids who have yet to develop the capabilities to fully understand and make autonomous decisions still retain the right to have their preferences engaged with and respected.^54^ For individuals with dementia with diminished autonomy, current values and preferences should not be ignored simply because the individual has lost the capacity to make fully autonomous decisions. We should still attempt to respect what is left of an individual's ability to make choices about their life.

The vast majority of individuals with dementia, such as the ones discussed in this paper, fall under the category of individuals warranting autonomy considerations—they are individuals with a wide range of partial autonomy who retain the ability to value. For these individuals, I argue, their remaining values should be respected as well as possible because they represent the best guide for what is left of their ability to self‐determine. As I have argued, individuals with moderate‐to‐severe dementia can retain the ability to value in many cases, even when they lack decision‐making capacity. This ability to value lays the starting point for their expression of autonomy, by enabling them to hold interests about what they care about and how they want their life to be led.^55^ However, many individuals with dementia who are incapable require those around them to implement such values, as they do not have the decision‐making capacity to make decisions for themselves due to the decline of cognitive faculties such as their verbal fluency and working memory. Thus, while an individual may have difficulty articulating their preferences amidst cognitive decline, the remaining individual's values should be interpreted by caregivers to the fullest extent realizable, and incorporated into decision‐making. This approach would apply to many individuals with moderate‐to‐severe dementia, to ensure that even though their autonomy is diminished, what remains of their ability to self‐determine is promoted through their remaining values.

For this group of individuals, instead of deferring to their advance directive due to their diminished autonomy, I contend that a substituted judgment approach to decision‐making is the best way to respect the current individual. Under this model, rather than advance directives being driving forces for a patient's care, decision‐making authority would be transferred to substitute decision‐makers, to determine what decision the incapable individual with dementia would want to make if they still had decision‐making capacity.^56^ While this model is often interpreted as a determination of what the individual would have wanted prior to losing capacity, dementia is different than other states of incapacity, such as a permanent vegetative state, because individuals with dementia can retain the capacity to value. Thus, this model is particularly advantageous in situations of dementia because it allows decision‐makers to account for the possibility of value changes occurring throughout the disease. Through communication and observation of the individual with dementia, decision‐makers can interpret what decision the individual would want *right now* if they had the cognitive capacity to express it.

The substituted judgment model would present challenges, such as the emotional burden placed on family members, and the difficulty in interpreting the values of an individual who is experiencing cognitive decline. However, these concerns would not be significant enough to sacrifice the chance of respecting what is left of an individual's diminishing autonomy. Even in the case of advance directives, caregivers must make very difficult interpretations about what an individual may have meant by their advanced wishes and determine when difficult directives concerning the end of life should be applied. Thus, it is not clear how much additional caregiver burden a substituted judgment model would generate. From a practical standpoint, the substituted judgment model would also require a closer examination of the range of decisions that family members can make on behalf of an incapable individual with dementia. In many countries, it is common for substitute decision‐makers to make decisions regarding the medical care of incapable individuals, including the termination of life‐extending measures and the initiation of palliative care.^57^ However, certain types of decisions, such as a substitute decision to implement euthanasia on behalf of an incompetent loved one, are generally prohibited.^58^ While close protections should be implemented for all substituted end‐of‐life decisions, it will be important to more thoroughly examine the principles upon which these distinctions are made.^59^ If, for instance, an individual with dementia is severely suffering and uttering that they do not want to continue living, should a substitute decision‐maker be permitted to initiate medical assistance in dying?

While this discussion has focused on individuals with dementia who retain an ability to value, a substituted judgment approach becomes more difficult for individuals with very advanced dementia, who retain little meaningful autonomy and have experienced cognitive and affective changes such that their current values can no longer be interpreted. In these situations, respecting an individual's remaining autonomy is nearly impossible, and it may seem justifiable to resort to the individual's priorly expressed preferences, either through the substitute decision‐maker or an advance directive. However, even in these situations, the current individual's interests should not be ignored. As Lauridsen illustrated, an advance directive cannot express an individual's autonomous interests if they no longer have any meaningful autonomy.^60^ An advance directive represents the interests of an individual's former self before they evolve through memory loss, cognitive decline, and affective changes. Individuals with severe dementia, even if they have lost the ability to value or their values can no longer be reliably interpreted, retain well‐being interests; they are individuals with daily experiences who have the potential to be harmed by the imposition of precedent autonomy. Thus, while advance directives can serve as tools to inform substitute decision‐makers in situations when an individual with dementia can no longer value, such directives should only be implemented if they align with the individual's present interests.

This approach to decision‐making in dementia, in which an individual's current wishes are prioritized when their values can no longer be interpreted, aligns with much of medical practice over the past 20 years. Until recently, the Royal Dutch Medical Association stated that individuals with advance directives for euthanasia in the Netherlands must confirm the directive prior to its administration to ensure that an individual's present wishes align with their prior directive.^61^ Empirical work has found that, historically, this is how advance directives have been treated by healthcare providers; individuals' current well‐being, informed by their present wishes, guides much of dementia care.^62^ This approach ensures that once an individual's autonomy can no longer be exercised, the well‐being of the current individual, who may be harmed by a past decision that contradicts their current interests, retains significant importance.^63^

However, as the influence of advance directives increases in several countries, there is a risk that individuals' current interests will begin to be neglected in favor of precedent autonomy. Recently, in the Netherlands, the Supreme Court acquitted a physician who sedated a semi‐resistant Alzheimer's disease patient in order to administer euthanasia, because the individual had requested euthanasia in her advance directive.^64^ Moreover, the Netherlands has changed its guidelines for euthanasia in incompetent patients, such that it is no longer required for an individual with dementia to affirm their previous directive. Although in the Dutch example, the patient was widely believed to be severely suffering such that their current desires seemed to align with their advance directive, this may change in the future as the influence of advance directives increases for states of incompetence. In Canada, the implementation of Medical Assistance in Dying for persons with dementia is gaining political traction, and several other countries, such as Colombia, Spain, and Belgium, are allowing such advanced requests.^65^ It is important that, as the authority of advance directives for dementia expands, we do not neglect the values and well‐being of the individuals in front of us.

## 8 CONCLUSIONS

As an individual with dementia loses memories, they may change their values because they have less access to past experiences. As I have argued, Jongsma's “choosing and losing” distinction does not explain why advance directives should retain moral authority in these cases. If individuals with dementia can retain the capacity to value, then the validity of extending past preferences to a later time in which their values may change is questionable. Rather than holding someone to their past preferences, respecting the diminishing autonomy of individuals with dementia requires giving caregivers the responsibility to interpret the individual's current values. Crucially, when making these interpretations, it is important to understand that what an individual wants now may be different from what they expressed previously. Caring for our vulnerable populations is an incredibly difficult and nuanced duty, but doing it right and in a way that honors the wishes of those individuals, is something that we must not overlook.

## Footnotes

1 Arvanitakis, Z., & Bennett, D. A. (2019). What is dementia? *JAMA*, *322*(17), 1728.

2 Ibid.

3 Nichols, E., Steinmetz, J. D., Vollset, S. E., Fukutaki, K., Chalek, J., Abd‐Allah, F., Abdoli, A., Abualhasan, A., Abu‐Gharbieh, A., Tayyaba Akram, T., Al Hamad, H., Alahdab, F., Alanezi, F. M., Alipour, V., Almustanyir, S., Amu, H., Ansari, I., Arabloo, J., Ashraf, T., … Vos, T. (2022). Estimation of the global prevalence of dementia in 2019 and forecasted prevalence in 2050: An analysis for the Global Burden of Disease Study 2019. *The Lancet Public Health*, *7*(2), 105–125.

4 Beauchamp, T. L., & Childress, J. F. (1994). *Principles of biomedical ethics* (4th ed.). Oxford University Press; Buchanan, A. E., & Brock, D. W. (1990). *Deciding for others: The ethics of surrogate decision‐making*. Cambridge University Press; Dworkin, R. (1993). *Life's dominion: An argument about abortion, Euthanasia, and individual freedom*. Knopf.

5 De Boer, M. E., Hertogh, C. M., Dröes, R. M., Jonker, C., & Eefsting, J. A. (2010). Advance directives in dementia: Issues of validity and effectiveness. *International Psychogeriatrics*, *22*(2), 201–208.

6 Ibid.

7 Ibid; Dresser R. (2017). On legalizing physician‐assisted death for dementia. *Hastings Center Report, 47*(4), 5–6.

8 Buchanan, A. E., & Brock, D. W., op. cit. note 4, p. 37.

9 Dworkin, op. cit. note 4.

10 Ibid.

11 Ibid.

12 Ibid.

13 Ibid: 224.

14 Ibid: 225.

15 Beauchamp & Childress, op. cit. note 4; Huang, Y., Cong, Y., & Wang, Z. (2022). Rethinking the precedent autonomy, current minimal autonomy, and current well‐being in medical decisions for persons with dementia. *Bioethical Inquiry*, *19*(1),163–175; Kollisch, D. O., Santulli, R. B., & Bernat, J. L. (2021). The limits of advance directives in maintaining autonomy in patients with advanced dementia. *American Journal of Medicine*, *134*(8), 963–967.

16 Dworkin, op. cit. note 4; Jaworska, A. (1999). Respecting the margins of agency: Alzheimer's patients and the capacity to value. *Philosophy & Public Affairs*, *28*(2), 105–138.

17 Firlik, A. D. (1991). Margo's logo, *JAMA*, *265*(2), 201.

18 Ibid: 201.

19 Dworkin, op. cit. note 4.

20 Lyreskog, D. M. (2021). Withering minds: Towards a unified embodied mind theory of personal identity for understanding dementia. *Journal of Medical Ethics*, *49*, 699–706.

21 Dresser, R. (1995). Dworkin on dementia: Elegant theory, questionable policy. *The Hastings Center Report*, *25*(6), 32–38.

22 Lyreskog, op. cit. note 20; Berghmans, R. (2000). Advance directives and dementia. *Annals of the New York Academy of Sciences*, *913*, 105–110.

23 Rhoden, N. K. (1988). Litigating life and death. *Harvard Law Review,102*(2), 375–446.

24 Lyreskog, op cit. note 20.

25 Walsh, E. (2020). Cognitive transformation, dementia, and the moral weight of advance directives. *The American Journal of Bioethics*, *20*(8), 54–64.

26 Here, I follow Jongsma, K. R., Sprangers, M. A. G., & van de Vathorst, S. (2016). The implausibility of response shifts in dementia patients. *Journal of Medical Ethics*, *42*(9), 597–600, in distinguishing between preferences, which are guided by values, and moment‐to‐moment “wishes.”

27 Jaworska, op. cit. note 16.

28 Jongsma, K. R., et al., op. cit. note 26; Jongsma, K. R., Kars, M. C., & Delden, J. J. M. (2019). Dementia and advance directives: Some empirical and normative concerns. *Journal of Medical Ethics*, *45*(2), 92–94; Jongsma, K. (2020). Losing rather than choosing: A defense of advance directives in the context of dementia. *The American Journal of Bioethics*, *20*(8), 90–92.

29 Jongsma, K. R., et al., op cit. note 26, p. 599.

30 Ibid.

31 Jongsma, op. cit. note 28, 90–92.

32 Ibid.

33 Ibid.

34 Jongsma, K. R., et al., op. cit. note 26.

35 Ibid.

36 Ibid.

37 Jaworska, A., op. cit. note 16.

38 Ibid.

39 Scheffler, S. (2010). *Equality and tradition: Questions of value in moral and political theory*. Oxford University Press.

40 Dementia can also impact other cognitive domains, such as an individual's affect and emotional responses (i.e., Cerejeira, J., Lagarto, L., & Mukaetova‐Ladinska, E. B. (2012). Behavioral and psychological symptoms of dementia. *Frontiers in Neurology, 3*(73), 1–21.) More work is required on the role that these cognitive changes may have on an individual's decision‐making and ability to value. However, it is unlikely that these affective changes would alter my overall characterization of individuals with dementia as “valuers.” While emotional responses can change during dementia, the ability to hold strong feelings toward decisions, which is necessary for some accounts of valuing, such as Scheffler's, persists. If anything, it is possible that affective changes may present another avenue for an individual's value set to change, by altering the sorts of experiences that an individual feels strongly toward and pursues.

41 Smebye, K. L., Kirkevold, M., & Engedal, K. (2012). How do persons with dementia participate in decision‐making related to health and daily care? A multi‐case study. *BMC Health Services Research*, *12*(241), 1–12.

42 de Boer, M. E., Hertogh, C. M., Dröes, R. M., Riphagen, I. I., Jonker, C., & Eefsting, J. A. (2007). Suffering from dementia**—**The patient's perspective: A review of the literature. *International Psychogeriatrics*,*19*(6), 1021–1039; Steeman, E., De Casterlé, B. D., Godderis, J., & Grypdonck, M. (2006). Living with early‐stage dementia: A review of qualitative studies. *Journal of Advanced Nursing, 54*(6), 722–738; Wolverson, E. L., Clarke, C., & Moniz‐Cook, E. D. (2015). Living positively with dementia: A systematic review and synthesis of the qualitative literature. *Aging & Mental Health*, *20*(7), 676–699.

43 Darby, R. R., & Dickerson, B. C. (2017). Dementia, decision‐making, and capacity. *Harvard Review of Psychiatry, 25*(6), 270–278.

44 Ibid.

45 Ibid; Leo, R. J. (1999). Competency and the capacity to make treatment decisions: A primer for primary care physicians. *Primary Care Companion to The Journal of Clinical Psychiatry*, 1(5), 131–141.

46 Zwijsen, S. A., van der Ploeg, E., Hertogh, C. M. (2016). Understanding the world of dementia. How do people with dementia experience the world? *International Psychogeriatrics*, *28*(7), 1067–77.

47 Thank you to an anonymous reviewer for raising the question of whether the ability to value would also require an individual to demonstrate the presence of a value *hierarchy* and explain why one value should take precedence over others. Within dementia, it is likely that an individual with the ability to value and hold preferences would maintain a hierarchy of values. For instance, the individual in Jaworska's example who expresses a preference to volunteer must hold her value of altruism above alternative values, such as personal comfort, which enables her to prefer volunteering over other alternatives. The ability to explain these value‐driven decisions is more demanding, requiring a further degree of decision‐making capacity. However, this ability is not required for us to at least be concerned about the possibility that incapable individuals' values have changed.

48 This is also the case with values that are “lost” due to cognitive decline. While losing previously held values produces an unchosen change in an individual's remaining set of values, the values that remain should still be respected, so long as the individual affirms them and accepts them as their own.

49 Jongsma, K. R., et al., op. cit. note 26, p. 599.

50 Beauchamp, T. L., & Childress, J. F. (2013). *Principles of biomedical ethics* (7th ed.). Oxford University Press.

51 Soofi, H. (2022). Respect for autonomy and dementia care in nursing homes: Revising Beauchamp and Childress's account of autonomous decision‐making. *Journal of Bioethical Inquiry*, *19*, 467–479.

52 Ibid.

53 Ibid.

54 Ibid.

55 Jaworska, op. cit. note 16.

56 Torke, A. M., Alexander, C. G., & Lantos, J. (2008). Substituted judgment: The limitations of autonomy in surrogate decision‐making. *Journal of General Internal Medicine*, *23*(9), 1514–1517; Jaworska, A. (2009). Advance directives and substitute decision‐making. *Stanford Encyclopedia of Philosophy.* Retrieved from https://plato.stanford.edu/archives/sum2017/entries/advance-directives/; Sulmasy, D. P., & Snyder, L. (2010). Substituted interests and best judgments: An integrated model of surrogate decision‐making. *JAMA, 304*(17), 1946–1947.

57 Government of Canada. (2023, November 23). *Palliative care: Preparing and deciding*. https://www.canada.ca/en/health-canada/services/health-services-benefits/palliative-care/preparing-deciding.html; General Medical Council. (2024). *Legal Annex*. https://www.gmc-uk.org/professional-standards/professional-standards-for-doctors/treatment-and-care-towards-the-end-of-life/legal-annex.

58 Government of Canada. (2023, February 3). *Consultations of physician‐assisted dying—Summary of results and key findings*. https://www.justice.gc.ca/eng/rp-pr/other-autre/pad-amm/p6.html.

59 With respect to advance directives, Van Delden, J. J. M. (2004). The unfeasibility of requests for euthanasia in advance directives. *Journal of Medical Ethics, 30*(5), 447–445 has asserted that there is a morally relevant difference between negative requests, such as the withdrawal of care, and positive requests, such as euthanasia, because positive directives impose a demanding moral request on another individual (i.e., the administering physician). This distinction requires further exploration in the context of substitute decision‐making.

60 Lauridsen, S., Folker A. P., & Anderson M. M. (2023). Why caregivers have no autonomy‐based reason to respect advance directives in dementia care. *Bioethics, 37*, 399–405.

61 Wijsbek, H., & Nys, T. (2022). On the authority of advance euthanasia directives for people with dementia: Reflections on a Dutch case. *Hastings Center Report, 52*(5), 24–31.

62 Kalis, A. van Delden, J., & Schermer, M. (2004). “The good life” for demented persons living in nursing homes. *International Psychogeriatrics, 16*(4), 429–439.

63 KNMG Federation Board. (2021, November 18). *KNMG guideline end‐of‐life decisions*. https://www.knmg.nl/actueel/publicaties/publications-in-english. As the KNMG guidelines state, in the case of euthanasia, individuals must also be observably suffering over an extended period of time, either through their behavior or their verbal communication, in order for directives to be implemented.

64 Wijsbek & Nys, op. cit. note 61, p. 26; Boffey, D. (2019, September 11). Dutch doctor acquitted in landmark euthanasia case. *The Guardian*.

65 Zandenbergen, R. (2024, February 13). Should dementia patients be able to make advance requests for medical assistance in dying? *CBC News*. https://www.cbc.ca/news/canada/ottawa/dementia-patient-maid-canada-request-rules-1.7101508

